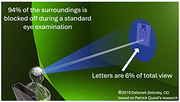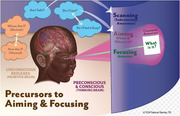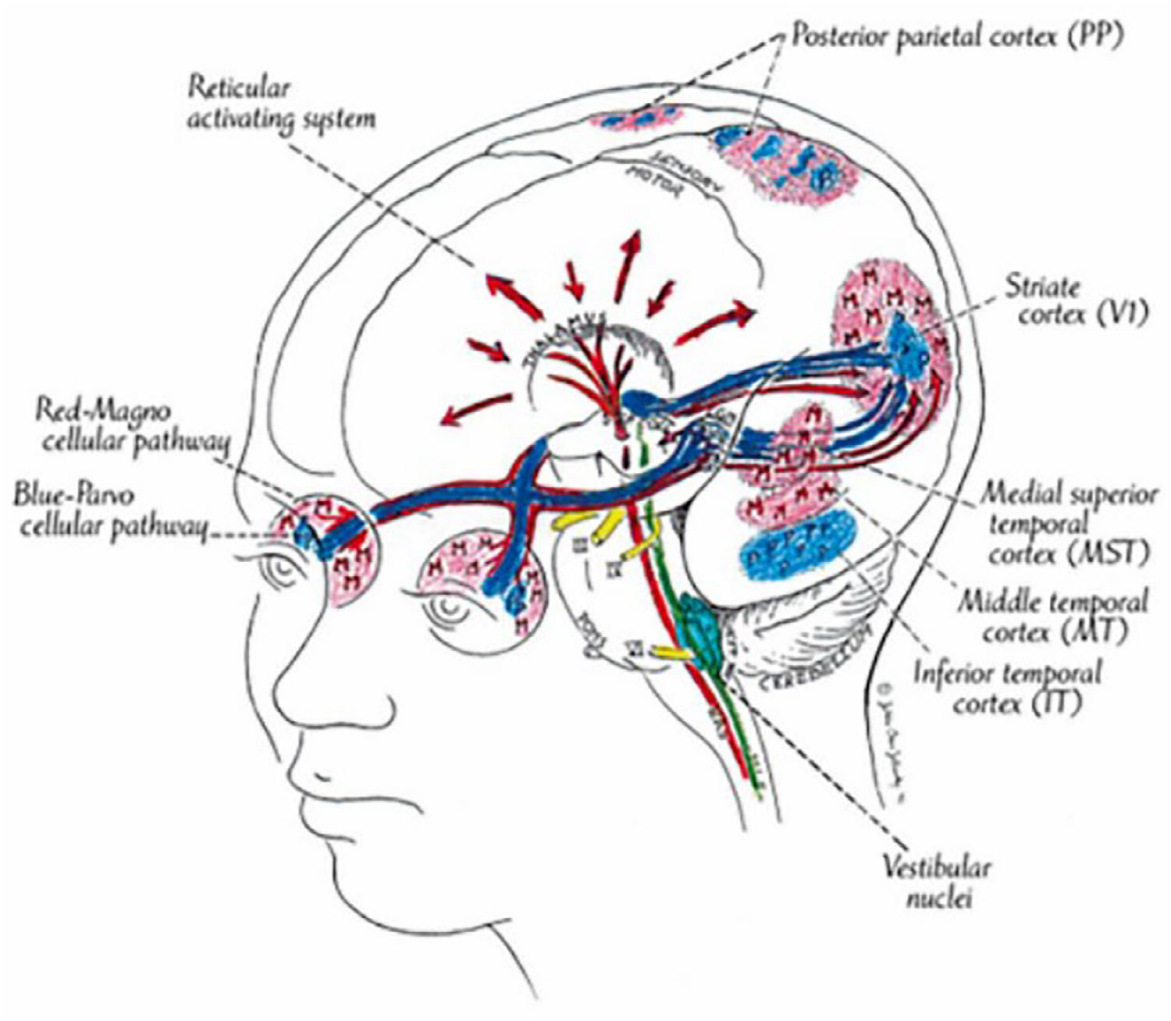# Future Possibilities of Enhancing Mild Cognitive Impairments by Resolving Eye/Ear Disconnects in Alzheimer's and TBI patients

**DOI:** 10.1002/alz70861_108704

**Published:** 2025-12-23

**Authors:** Deborah Zelinsky, Delia Cabrera DeBuc

**Affiliations:** ^1^ The Mind‐Eye Institute, Northbrook, IL USA; ^2^ iScreen 2 Prevent, LLC, Pembroke Pines, FL USA

## Abstract

**Background:**

Alzheimer’s Disease (AD) and Traumatic Brain Injury (TBI) are significant causes of disability in the USA, with millions affected and high treatment costs. Research has identified disrupted connections between auditory and visual sensory pathways in both conditions, leading to impairments in balance, sleep, posture, motor function, sensory integration (including visual‐vestibular), and emotional regulation. These disruptions cause symptoms such as spatial disorientation, dizziness, comprehension difficulties, and Post Traumatic Stress Disorder (PTSD)‐related issues such as anxiety, panic attacks, and hypervigilance. Even mild TBIs can result in structural brain damage, chronic headaches, and progressive cognitive decline. Furthermore, multiple TBIs significantly increase the risk of developing Alzheimer’s. The early detection of mild cognitive impairments (MCI) is critical to prevent the further cognitive decline. Standard physical and occupational therapies often fail to adequately address the chronic symptoms associated with AD, TBI and PTSD leaving patients with persistent difficulties and a diminished quality of life.

**Method:**

A proposed solution includes a double‐blind study to assess the combined benefits of neuro‐optometric rehabilitation and neuroplasticity training compared to standard corrective interventions.

**Result:**

Research using retinal mapping could lead to significant advancements in early Alzheimer’s, TBI and PTSD care, improving patients’ quality of life and offering new treatment pathways. Success would also establish a replicable model for nationwide implementation, particularly in healthcare settings, where these conditions are prevalent

**Conclusion:**

Emerging evidence supports a promising approach for improving sensory function and reducing MCI, TBI and PTSD symptoms via retinal stimulation. However, a lack exists of rigorous, controlled studies to scientifically validate its effectiveness.